# Hedgehog-interacting protein is highly expressed in endothelial cells but down-regulated during angiogenesis and in several human tumors

**DOI:** 10.1186/1471-2407-4-43

**Published:** 2004-08-04

**Authors:** Catherine L Olsen, Pin-Pin Hsu, Jens Glienke, Gabor M Rubanyi, Alan R Brooks

**Affiliations:** 1 Department of Gene Therapy, Berlex Laboratories, Inc., Richmond, CA 94806, USA; 2 SBU DG&RP, Schering AG, Berlin, Germany; 3 Present location: Exelixis, Inc., South San Francisco, CA 94083, USA

## Abstract

**Background:**

The Hedgehog (Hh) signaling pathway regulates a variety of developmental processes, including vasculogenesis, and can also induce the expression of pro-angiogenic factors in fibroblasts postnatally. Misregulation of the Hh pathway has been implicated in a variety of different types of cancer, including pancreatic and small-cell lung cancer. Recently a putative antagonist of the pathway, Hedgehog-interacting protein (HIP), was identified as a Hh binding protein that is also a target of Hh signaling. We sought to clarify possible roles for HIP in angiogenesis and cancer.

**Methods:**

Inhibition of Hh signaling by HIP was assayed by measuring the induction of Ptc-1 mRNA in TM3 cells treated with conditioned medium containing Sonic hedgehog (Shh). Angiogenesis was assayed in vitro by EC tube formation on Matrigel. Expression of HIP mRNA was assayed in cells and tissues by Q-RT-PCR and Western blot. HIP expression in human tumors or mouse xenograft tumors compared to normal tissues was assayed by Q-RT-PCR or hybridization of RNA probes to a cancer profiling array.

**Results:**

We show that Hedgehog-interacting protein (HIP) is abundantly expressed in vascular endothelial cells (EC) but at low or undetectable levels in other cell types. Expression of HIP in mouse epithelial cells attenuated their response to Shh, demonstrating that HIP can antagonize Hh signaling when expressed in the responding cell, and supporting the hypothesis that HIP blocks Hh signaling in EC. HIP expression was significantly reduced in tissues undergoing angiogenesis, including PC3 human prostate cancer and A549 human lung cancer xenograft tumors, as well as in EC undergoing tube formation on Matrigel. HIP expression was also decreased in several human tumors of the liver, lung, stomach, colon and rectum when compared to the corresponding normal tissue.

**Conclusion:**

These results suggest that reduced expression of HIP, a naturally occurring Hh pathway antagonist, in tumor neo-vasculature may contribute to increased Hh signaling within the tumor and possibly promote angiogenesis.

## Background

The Hedgehog family of genes encodes secreted signaling molecules that regulate cell proliferation and cell fate determination. In mammals, there are three such genes: Sonic hedgehog (*Shh*), Indian hedgehog (*Ihh*), and Desert hedgehog (*Dhh*). All three Hedgehog (Hh) proteins function by binding to the transmembrane receptor, Patched-1 (Ptc-1), leading to the de-repression of the membrane-bound inhibitor, Smoothened [1,2]. This results in activation of the transcription factor Gli-1, which induces expression of target genes that include Ptc-1 and Gli-1 itself. The increase in expression of Ptc-1 may limit the range of action of Hh by sequestering it at the surface of Hh-responsive cells [3].

Hedgehog-interacting protein (HIP) was discovered by screening a mouse cDNA expression library for proteins that bound to Shh [4]. HIP binds all three Hh proteins with an affinity equal to that of Ptc-1, and in mouse embryos it is expressed in cells adjacent to those expressing Shh, positioning it appropriately for in vivo interactions. Ectopic expression of Shh leads to ectopic HIP expression, indicating that HIP is a transcriptional target of Hh signaling [4]. Transgenic mice that overexpress HIP in the endochondral skeleton displayed a phenotype similar to that of Ihh knockout mice, consistent with an inhibitory role for HIP in Hh signaling [4]. Although it has been shown that overexpression of HIP in cells making Shh reduced the amount of Shh secreted into the media [5], no data has been published specifically demonstrating that expression of HIP in responding cells inhibits the activation of the Shh signaling pathway.

During gastrulation in the mouse, Ihh is secreted by the endoderm and is sufficient to activate hematopoiesis and vasculogenesis [6]. In addition to its role in developmental processes, Shh was shown to induce angiogenesis in a murine corneal angiogenesis model, probably through the induction of the angiogenic factors VEGF, Ang-1, and Ang-2 [7]. Moreover, inhibition of Shh in the ischemic hindlimb of mice through the use of a neutralizing antibody inhibits endogenous angiogenesis [8]. Hh is also required for normal angiogenesis in the murine yolk sac, as Ihh^-/- ^mice can initiate vasculogenesis and hematopoiesis but are defective in vascular remodeling to form blood vessels [9]. In the murine cornea, fibroblasts were identified as the Shh-responsive cells, while endothelial cells in the corneal neovessels, as well as human umbilical vein endothelial cells and microvascular endothelial cells cultured *in vitro*, were unable to respond to Shh, even though they express the receptor Ptc-1 [7].

Misregulation of the Hh signaling pathway has been implicated in several different types of cancer, including basal cell carcinomas (BCCs), medullablastomas, and gliomas (reviewed in [10]. Mutations in Ptc-1, which lead to constitutive activation of the Hh pathway, are the underlying factor in basal cell nevus syndrome, a familial condition characterized by a predisposition to BCC development [11]. In addition, Gli-1 was originally identified as a gene overexpressed in a human glioma line [12]. Recently, it was reported that Hh pathway activity is upregulated in digestive tract, pancreatic, and small-cell lung tumors and is required for the growth of these tumors [13-15].

Here we show that overexpression of HIP in cultured mouse Leydig cells inhibits the ability of these cells to respond to exogenous Shh, confirming that HIP can block Hh signaling when expressed in the same cells as the receptor Ptc-1. We have also shown that HIP is expressed predominantly in vascular endothelial cells and is downregulated in HUVEC during *in vitro *angiogenesis. Strikingly, HIP mRNA levels were decreased or even absent in several types of human tumors, as well as in highly vascularized human tumors grown in nude mice. Taken together, these results indicate a correlation between angiogenesis and decreased expression of HIP, lending support to the role of HIP as a naturally occurring regulator of Hh signaling and neovessel formation in the adult organism.

## Methods

### Conditioned media production

Adherent cultures of 293-EBNA cells were transfected with a Shh-pCDNA3 expression plasmid kindly provided by Dr. Pao-Tien Chuang (UCSF) using LipofectAMINE 2000 and OptiMem reduced-serum medium (Invitrogen). Three to five hours after transfection, cells were re-fed with serum-containing growth medium, and three or four days later conditioned medium was collected and filtered through 0.2-μm cellulose acetate filters.

### Endothelial cell tube formation assays

For RNA isolation, human aortic endothelial cells (HAEC) were cultured in 12-well plates with or without Matrigel (BD Biosciences) for 16 hours, then cells were lysed, digested with proteinase K, and total RNA was extracted using the RNeasy Mini Kit (Qiagen). HIP expression was assayed by real-time quantitative reverse transcriptase PCR (Q-RT-PCR) and normalized to 18s rRNA (see below).

### Transfection and conditioned medium assays

The mouse testicular epithelial cell line, TM3, was grown in complete medium (DMEM/F12 with 2.5% FBS, 5% horse serum, 15 mM Hepes, and 4 mM glutamine) at 37°C and 5% CO_2_. Cells were transfected in 12-well or 24-well plates at 80–90% confluence using LipofectAMINE 2000 transfection reagent and OptiMem reduced-serum medium (Invitrogen). For conditioned medium (CM) assays, cells were transfected with the plasmid MycHIP-pCDNA3 (gift of Dr. Pao-Tien Chuang, UCSF), which encodes full-length mouse HIP, or empty vector (mock). Mock or Shh CM was added to cells 36 hours after transfection, then RNA was harvested 36–48 hours later.

### Collection of mouse tissues

PC3 human prostate cancer cells were implanted subcutaneously into male nude mice (strain nu/nu), and on day 39 the resulting tumors were excised, weighed, placed on dry ice, then stored at -80°C prior to RNA isolation. A549 lung cancer cells were implanted subcutaneously into female nude mice and harvested as described above on day 9. Normal livers or skin from 5- to 6-week-old male or 4- to 5-week-old female nude mice were excised immediately following cervical dislocation and frozen in liquid nitrogen, then stored at -80°C.

### RNA isolation

RNA was extracted from cultured cells using the RNeasy kit (Qiagen), including on-column DNase I digestion, according to the manufacturer's instructions. To extract RNA from frozen normal mouse tissues and tumors, hard-frozen tissue samples of 100–300 μg were pulverized over dry ice, placed in denaturing buffer, and disrupted using a Mixer Mill (Retsch) or rotor-stator homogenizer (Omni International). RNA was isolated from the homogenate either by phenol-chloroform extraction followed by precipitation with isopropanol and washing with 70% ethanol (ToTALLY RNA kit, Ambion) or by using the RNeasy kit (Qiagen). All RNA samples were treated with DNase I (Qiagen) prior to quantitative RT-PCR. Concentrations of RNAs were determined by reading the absorbance at 260 nm.

### Quantitative RT-PCR

Quantitative RT-PCR was performed using the ABI PRISM™ 7700 Sequence Detection System and TaqMan™ chemistry. Reactions contained 0.3 μM of each primer, 0.1 μM probe, 0.25 U/ml MultiScribe reverse transcriptase, 0.4 U/ml RNase inhibitor, and 1x PCR master mix (Applied Biosystems). Primers and probes used to quantitate the mRNA level of various genes were as follows: mouse Ptc-1 (forward primer 5'GCCAATGGCCTAAACCGACT, reverse primer 5'AAACCGGACGACACTTGGAG, probe 5'6FAM-CCCACTCCTTCGCCTGAGCCG-TAMRA), mouse HIP (forward primer 5'CCACTGACCTCCGATTGCTC, reverse primer 5'TGCAGCAGCACTTGCCAG, probe 5'6FAM-CGGCTCTGTCGAAACGGCTACTACACC-TAMRA), mouse vWF (forward primer 5'CCGGAAGCGACCCTCAGA, reverse primer 5'CGGTCAATTTTGCCAAAGATCT, probe 5'6FAM-TGGCCTCTACCAGTGAGGTTTTGAAGTACACAC-TAMRA), mouse CD146 (forward primer 5'GGGCCTCAGGCAACTTCA, reverse primer 5'TTGGTGCACACGGAAAATCA, probe 5'6FAM-CTCCTTGTGAATCAAAAACCAGTCCACTTGG-TAMRA), human HIP (forward primer 5'CCCACACTTCAACAGCACCA, reverse primer 5'GCACATCTGCCTGGATCGT, probe 5'6FAM-CCCCGAAGTGTTTGCTCATGGGCT-TAMRA), human vWF (forward primer 5'TGAAGTATGCGGGCAGCC, reverse primer 5'GCGGTCGATCTTGCTGAAG, probe 5'6FAM-CCTCCACCAGCGAGGTCTTGAAATACAC-TAMRA). Mouse and human GAPDH and ribosomal RNA probe and primer sets were purchased from Applied Biosystems. RNA quantities were determined from a standard curve of serially diluted total cellular or tissue RNA run in parallel with each set of reactions. Standard curves had a slope between -3.1 and -3.3 and correlation coefficients of 0.98 or greater.

### Western blot

Total cell lysates were made by lysing cells in 2% SDS, 10% glycerol, and 0.063 M Tris-HCl (pH 6.8) containing a cocktail of protease inhibitors (Pierce). Lysates were heat denatured at 95°C for 10 minutes, passed through a 26G needle to shear DNA, and centrifuged at 10,000 g for 30 minutes to remove insoluble material. Protein concentrations were determined using the BCA assay (Pierce). Conditioned medium samples were diluted 1:1 in Laemmli sample buffer (Sigma). 50 μg of cell lysate or 20 μl of conditioned medium/sample buffer was resolved on 4–15% polyacrylamide gels (BMA) and transferred to nitrocellulose (Invitrogen). Even loading of protein samples was verified by staining with Ponceau S. After overnight blocking with 5% nonfat dry milk, 0.05% Tween-20 in phosphate-buffered saline, blots were incubated with antibodies against Shh (sc-1194, Santa Cruz), Myc tag (Invitrogen), or HIP (provided by Dr. Pao-Tien Chuang, UCSF). Antibody-antigen complexes were visualized using horseradish peroxidase-conjugated secondary antibodies (Jackson ImmunoLabs, Santa Cruz) and SuperSignal West Pico chemiluminescent substrate (Pierce).

### Hybridization of RNA probes to cancer array

Linearized templates containing a 716-bp fragment of human HIP cDNA (bases 521–1236 of human HIP coding sequence) or a 777-bp fragment of human vWF cDNA (bases 7301–8077 of human vWF coding sequence) were radiolabelled with ^32^P-dUTP by *in vitro *transcription using the MAXIscript kit (Ambion). The array was pre-hybridized in Clontech ExpressHyb solution with 100 μg/ml boiled, sheared salmon testis DNA (SS-DNA) for 2–12 hours at 68°C with rotation. Pre-hybridization solution was replaced with fresh hybridization solution with SS-DNA, plus a total of 1.5–1.7 × 10^7 ^cpm of ^32^P-labelled HIP or vWF probe. Hybridization of the probe proceeded for 16–18 hours at 68°C, followed by four washes in 2X SSC/0.5% SDS at 68°C, one wash in 0.2X SSC/0.5% SDS at 68°C, and one wash in 2X SSC at room temperature. The array was exposed to a phosphor-screen for 24–72 hours and scanned in a Storm phosphor-imager at a resolution of 50 μm. After hybridization to the HIP probe, the same array was stripped by boiling in 0.5% SDS and re-scanned to verify the absence of residual probe before hybridization to the vWF probe. Relative levels of HIP and vWF expression were determined using ImageQuant software (Molecular Dynamics).

### Statistical analysis

All results are expressed as mean ± standard deviation unless otherwise noted. Statistical significance of differences was determined using a two-tailed Student's t-test.

## Results

### HIP is highly expressed in endothelial cells

**Figure 1:**
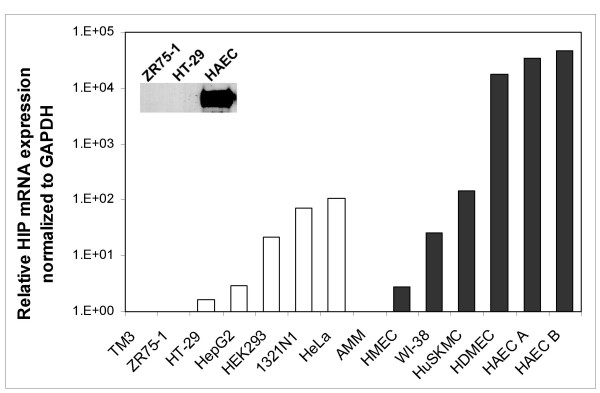
**Comparison of HIP expression in different cell types. **Total RNA from each cell type was assayed for HIP and GAPDH mRNA levels by Q-RT-PCR. Relative HIP mRNA expression was normalized to GAPDH and plotted on a log scale. Open bars, immortalized or cancer cell lines; filled bars, normal cells. TM3, mouse testicular epithelial cell line; ZR75-1, human breast cancer cell line; HT-29, human colon cancer cell line; HepG2, human liver cancer cell line; HEK293, human kidney cancer cell line; 1321N1, human brain cancer cell line; HeLa, human cervical cancer cell line; AMM, activated mouse macrophage; HMEC, normal human mammary epithelial cells; WI-38, normal human lung fibroblasts; HuSKMC, normal human embryonic skeletal muscle cells; HDMEC, normal human dermal microvascular endothelial cells; HAEC A and HAEC B, normal human aortic endothelial cells from two different donors (obtained from Cascade Biologics and Clonetics, respectively).

Ptc-1 and Smo mRNA are expressed in human aortic endothelial cells (HAEC) at levels comparable to that of GAPDH, as determined by comparing the threshold cycle numbers obtained by quantitative RT-PCR (Q-RT-PCR) (data not shown). Thus HAEC express components of the Hh pathway that are known to mediate a response to Hh ligands. However, when HAEC or human umbilical vein endothelial cells (HUVEC) were treated with conditioned medium containing functional Shh, the Hh pathway was not activated as determined by measuring the levels of mRNA for Ptc-1 and Gli-1, two genes known to be responsive to Hh signaling (data not shown). Shh mRNA levels in HAEC and HUVEC were near the limits of detection by the Q-RT-PCR assay and could therefore be estimated at less than 10^-3 ^copies per cell based on the typical sensitivity of this assay. Hedgehog-interacting protein (HIP) binds Hh proteins with an affinity equal to that of Ptc-1 and can function as an antagonist of Ihh signaling in vivo [4]. Thus we hypothesized that HIP may be expressed in EC and function to block Hh signaling. Indeed, we found that HAEC and HDMEC expressed amounts of HIP mRNA similar to that of GAPDH, as measured by Q-RT-PCR. Endothelial cell-predominant expression of HIP was demonstrated by Q-RT-PCR analysis of a variety of human and mouse primary cell strains and immortalized cell lines and human cancer cell lines representing different tissues. When normalized to the level of GAPDH mRNA, the level of HIP mRNA was 100- to 10,000-fold higher in vascular endothelial cells than in the other cell types examined (Figure 1). Expression of HIP protein in HAEC, but not in ZR75-1 or HT-29, was confirmed by Western blot (Figure 1, inset). The observation that HIP mRNA is expressed at a high level in human endothelial cells suggests that HIP may inhibit Hh signaling in these cells and may explain why these cells are unresponsive to Shh.

### HIP inhibits Ptc induction by Shh

**Figure 2:**
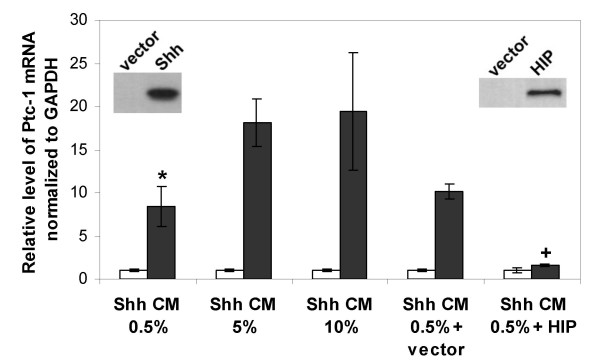
**Expression of HIP in responding cells blocks Ptc-1 induction by Shh. **Untransfected mouse TM3 cells (first three pairs of columns) or TM3 cells transfected with empty vector or a vector expressing mouse HIP (last two pairs of columns) were exposed to Shh conditioned media at the indicated concentrations (% v/v in normal growth media) for two days, after which Ptc and GAPDH mRNA levels were measured by Q-RT-PCR. Ptc-1 mRNA level was then normalized to that of GAPDH. Values shown for Shh conditioned media treatment (filled bars) are normalized to those obtained with mock conditioned media (open bars). Results shown are the mean of three experiments performed in duplicate. Error bars show standard deviation. The decrease in normalized Ptc mRNA level for mock- vs. Shh-treated (*) and for HIP- vs. vector-transfected (+) cells was statistically significant (p < 0.005, two-tailed Student's t-test). Left inset, Western blot showing Shh protein expression in conditioned media from Shh-transfected 293-EBNA cells. Right inset, Western blot showing HIP protein expression in conditioned media from HIP-transfected TM3 cells.

Conditioned media from 293 cells co-transfected with Shh and full-length (membrane-anchored) HIP contained a reduced amount of Shh and had a reduced ability to induce differentiation in C3H10T1/2 cells compared to cells transfected with Shh alone, demonstrating that HIP can sequester Shh [5]. However, there is no direct evidence that cells in which HIP is overexpressed are less responsive to Shh. In order to test this directly, 293-EBNA cells were transfected with a Shh expression plasmid. Western blot analysis demonstrated the presence of Shh protein in the conditioned medium from these cells (Figure 2, inset left). Exposure of TM3 mouse Leydig cells, a cell line with no detectable endogenous HIP mRNA (Figure 1), to increasing amounts of conditioned media containing Shh resulted in induction of the Shh pathway, as measured by a dose-dependent increase in the amount of endogenous Ptc-1 mRNA (Figure 2). When TM3 cells were transfected with a plasmid expressing full-length, N-terminally Myc-tagged mouse HIP, the induction of Ptc-1 mRNA by Shh was reduced by 79 percent (p < 0.05; Fig. 2), demonstrating that HIP functions as an inhibitor of the Shh pathway when expressed in the responding cells. Expression of HIP in transfected TM3 cells was verified by Western blot using a Myc antibody (Fig. 2, inset right).

### HIP is downregulated in endothelial cells during tube formation in vitro

**Figure 3:**
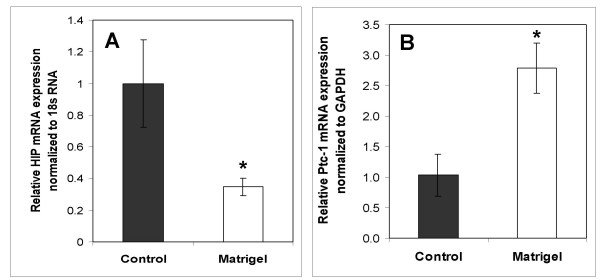
**HIP mRNA expression under *in vitro *angiogenic conditions. A**, HIP or **B**, Ptc-1 expression in HAEC cultured on plastic (control, filled bar) or on Matrigel (open bar). RNA was harvested from cells 16 hours after seeding on plastic or Matrigel, when tubes had fully formed on Matrigel, and assayed for HIP and 18s rRNA, or Ptc-1 and GAPDH mRNA, by Q-RT-PCR. The mean data are plotted as relative HIP mRNA normalized to 18s rRNA, or Ptc-1 mRNA normalized to GAPDH mRNA, and are the average of three (HIP) or two (Ptc-1) independent experiments performed in triplicate. Error bars show standard deviation. Differences in normalized HIP or Ptc-1 expression were significant for control vs. Matrigel (*p < 0.05, two-tailed Student's t-test).

Shh has been shown to induce angiogenesis [7], and HIP is able to antagonize the Shh pathway (Figure 2). Therefore, we tested whether HIP expression is downregulated in EC during tube formation, an essential step in the angiogenic process. Expression of HIP mRNA in HAEC that had formed tubes on Matrigel as measured by Q-RT-PCR was 2.9-fold lower (p < 0.05) than in the same cells cultured in standard plastic dishes (Figure 3A). To investigate whether there was a coordinated downregulation of Hh-regulated genes during tube formation on Matrigel, the mRNA level of Ptc-1, a known Hh-responsive gene, was measured. In contrast to HIP, levels of Ptc-1 mRNA increased 2.5-fold (p < 0.05) in cells on Matrigel compared to plastic (Figure 3B).

### HIP is downregulated in human tumors compared to normal tissues

Angiogenesis is a common feature of tumor growth (reviewed in [16,17]. It is conceivable that HIP is downregulated in this process, similar to the reduction we observed in endothelial cells during tube formation. Therefore, we measured HIP mRNA levels in a variety of tumor samples. Tumor and corresponding normal tissue RNA samples (normal and tumor tissues were from different individuals) were obtained from two different commercial sources (Ambion and BioChain), and the endothelial cell marker vWF was used to normalize HIP expression to the number of endothelial cells present in the tumors. In all tissue and tumor RNA samples assayed, vWF mRNA was detectable. In both pairs of liver tissue examined, HIP mRNA was expressed in normal tissue but was undetectable in the tumor (Table 1). One of two kidney tumor-normal pairs showed no difference in normalized HIP expression, while the other showed a 5.9-fold reduction of normalized HIP in tumor relative to normal tissue. Of the two breast tissue pairs, one had undetectable levels of HIP in both normal and tumor tissues (data not shown), while the other showed a 3.7-fold decrease in HIP expression in tumor compared to normal. In one lung tissue pair, vWF levels in normal tissue were over 900-fold lower than in the tumor, such that normalization of HIP to vWF resulted in an apparent 30-fold increase in HIP in tumor compared to normal tissue. In the other lung tissue pair, normalized HIP was 8.1-fold lower in tumor than in normal tissue.

To further investigate the frequency of the change in HIP expression in human tumors, a cancer profiling array (Clontech) containing cDNA from 154 tumor and corresponding normal tissues from individual patients was used to compare the expression of HIP in a larger set of samples. A ^32^P-labelled HIP probe corresponding to bases 521–1236 of human HIP cDNA was hybridized to the array, and relative levels of HIP expression were quantitated on a phosphoimager. HIP expression levels were normalized to the expression of the endothelial marker vWF, by re-probing the same array with a fragment of the human vWF cDNA, as different tumors and normal tissues may contain different degrees of vascularization. In liver, stomach, colon, and rectum, all or most of the paired samples showed a reduction of HIP in tumor compared to normal tissue (Table 2). In total, 28 samples had a decrease in the tumor, while only 3 samples showed a slight increase (1.3- to 1.7-fold), and 2 samples showed no change. The lung samples did not show a consistent patten of HIP mRNA expression. Five had a decrease of HIP mRNA in the tumor, while four had an increase and one showed no change. The remaining sets of paired normal and tumor tissues, including samples from breast, ovary, vulva, uterus, cervix, prostate, testis, thyroid, skin, bladder, small intestine, and pancreas, showed no marked difference in HIP expression between normal and tumor, or had undetectable HIP expression in both normal and tumor.

### HIP expression is very low or undetectable in xenograft tumors

**Figure 4:**
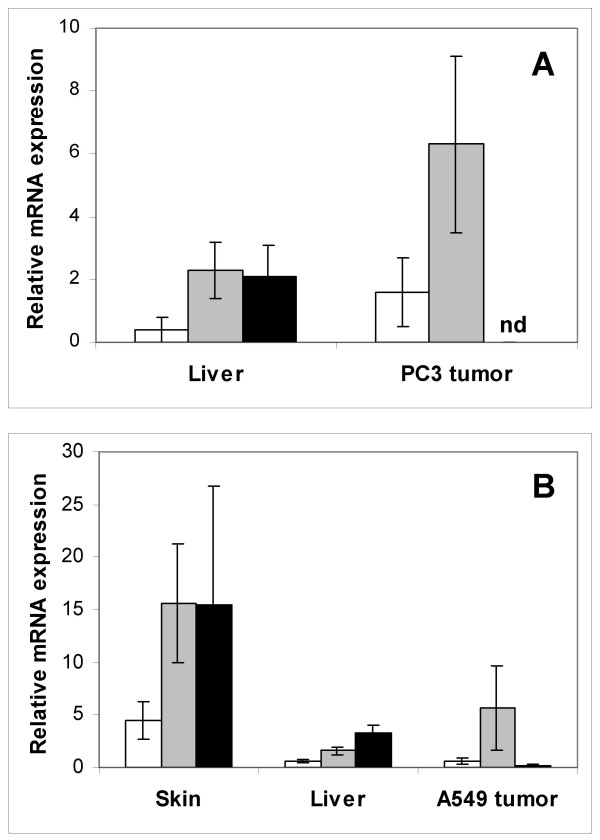
**HIP mRNA expression in normal mouse tissues vs. PC3 and A549 tumors. **100 ng of RNA extracted from **A**, normal livers or PC3 human prostate xenograft tumors from nude mice, or **B**, normal livers, normal skin, or A549 tumors from nude mice, were assayed for vWF, CD146, and HIP mRNA expression by Q-RT-PCR. The vWF (white bars), CD146 (gray bars), and HIP (black bars) mRNA levels were normalized to the GAPDH mRNA level in each RNA sample and are presented as the mean with standard deviation (**A**, N = 3 for normal liver, N = 8 for PC3 tumor; **B**, N = 5 for normal liver, N = 5 for normal skin, N = 3 for A549 tumor). nd = not detectable (no signal from Q-RT-PCR assay after 40 cycles).

Mouse xenograft tumors resulting from transplantation of PC3 (human prostate cancer cell line) or A549 (human lung cancer cell line) cells into nude mice are known to have high levels of neovascularization as a result of angiogenesis, which is required for their continued growth [18,19], so we hypothesized that HIP expression would be lower in these tumors than in normal tissues that also contain normal vessels. RNA extracted from PC3 or A549 tumors weighing an average of 350 mg that were excised 39 (PC3) or 9 (A549) days after subcutaneous implantation, or from liver or skin from non-implanted nude mice, was assayed for murine HIP, vWF, CD146, and GAPDH mRNA by Q-RT-PCR. The Q-RT-PCR assay for murine HIP is species-specific, i.e. does not detect human HIP mRNA (data not shown). Therefore, any HIP mRNA detected using this assay must be from host mouse cells present in the tumor, not from the human tumor cells themselves. Murine rather than human HIP was assayed because tumors have been shown to incorporate vasculature from the surrounding vessels [20-22] and there is evidence that tumors can recruit endothelial and hematopoietic precursor cells [23] to form their own vasculature. RNA from tumors and normal liver and skin contained measurable amounts of the murine endothelial cell markers vWF and CD146, indicating the presence of endothelial cells (Figure 4). For the PC3 xenograft experiment, the levels of vWF and CD146 mRNA normalized to GAPDH were actually higher in the tumors than in the normal liver, consistent with the highly vascularized nature of these tumors. In contrast, mouse HIP mRNA was abundant in all liver and skin samples examined but was 16- to 30-fold lower in A549 tumors and undetectable in any of the PC3 tumor samples (Figure 4A). These data demonstrate that although the tumors contained endothelial cells, they expressed markedly reduced or undetectable amounts of HIP mRNA.

## Discussion

Our demonstration that HIP functions to inhibit Ptc-1 upregulation in mouse testicular epithelial cells (TM3) exposed to Shh-containing conditioned media is the first direct evidence to date that Shh signaling is attenuated in cells that express full-length, membrane-bound HIP. The decrease in Ptc-1 induction caused by transfection with HIP is consistent with previous reports indicating that HIP can bind and reduce the availability of Shh and presumably prevent it from signaling through Ptc-1 [4,5]. In human aortic endothelial cells (HAEC), which express high levels of HIP, the addition of Shh-containing conditioned medium did not cause induction of Ptc-1, even though these cells express the receptor components Ptc-1 and Smo. In addition, others have shown that although Shh induces angiogenesis in a mouse model of hindlimb ischemia, it has no effect on endothelial cell proliferation or migration in cell culture [7]. Our finding that vascular EC express abundant amounts of HIP mRNA may explain the inability of these cells to respond to Shh. An analysis of various human cell lines and primary cells indicated that HIP is absent or expressed at low levels in other cell types, suggesting that in adults HIP is expressed primarily in EC. These results are supported by gene chip data analysis of more than 30 normal human tissues showing that HIP is most highly expressed in blood vessels or in vascular-rich tissues such as liver, lung, brain, and pancreas (data not shown). These results suggest a role for HIP in the normal function of blood vessels.

Several lines of evidence support the hypothesis that HIP expression is decreased in EC participating in angiogenesis. Firstly, we describe that HIP is downregulated in HAEC forming tubes on Matrigel. In contrast, Ptc-1 mRNA levels were increased in EC forming tubes on Matrigel, suggesting that the decrease in HIP mRNA under these conditions does not reflect a general downregulation of Hh-responsive genes. Given that transcription of HIP and Ptc-1 are both activated by Hh signaling, it is likely that the decrease in HIP expression in EC on Matrigel is mediated by a pathway independent of Hh signaling. Secondly, we have observed that HIP mRNA levels are decreased in human tumors and xenograft human tumors grown in mice, situations in which angiogenesis occurs. Previous studies have demonstrated a requirement for Shh in angiogenesis in the ischemic mouse limb and embryonic yolk sac [8,9]. The pro-angiogenic activity of exogenously supplied Shh protein has been attributed to the induction of factors such as VEGF and angiopoietin in fibroblasts [7]. Our data suggest the possibility that in situations where HIP expression is downregulated, Hh may also act directly on endothelial cells.

The Shh pathway has been implicated in several different types of cancer, where upregulation of signaling, associated with mutations in Patched or amplification of Gli, is often the suspected cause of malignancy [11,12,24-26]. In at least one type of human cancer, basal cell carcinoma (BCC), HIP upregulation has been demonstrated [27,28], probably as a result of dysregulation of the Shh signaling pathway. More recently it has been reported that Hh pathway activity is activated in a wide variety of digestive tract tumors by overexpression of the Hh ligand, and is essential for tumor growth [13]. Aberrant expression of Shh and activation of Hh signaling was also demonstrated to occur in pancreatic cancer [14] and small cell lung cancer [15]. Hh ligands secreted by tumor cells have the potential to induce Hh pathway activation in nearby non-tumor cells, including fibroblasts and endothelial cells. Our observation that HIP mRNA expression decreases in several human tumor types relative to normal tissues suggests that modulation of HIP expression may also promote increased Hh signaling in certain tumors and contribute to cancer progression. However, since expression of HIP is known to be induced by activation of the Hh pathway, an alternative interpretation is that a reduced level of HIP mRNA in tumors is indicative of reduced Hh signaling, as suggested by Hu et al. [29]. Our finding that, in endothelial cells in Matrigel, HIP mRNA levels decrease while Ptc-1 levels increase demonstrates that a mechanism other than Hh signaling can modulate the expression of these Hh target genes. Thus, the down-regulation of HIP in tumors may be unrelated to Hh pathway activity. The precise mechanism of HIP regulation by the Hh pathway in the context of tumors remains to be elucidated by future studies. Among the tumor types represented on the Clontech Cancer Profiling Array, liver and several areas of the digestive tract (stomach, colon, rectum) exhibited consistent downregulation of HIP in the tumor relative to normal tissue. Tumors from other tissues, including breast, testis, and kidney, did not show a significant change in HIP expression from the normal tissue, suggesting that this phenomenon may be restricted to certain tumor types. Differences in the degree of vascularization of the various tumors may also explain why a decrease in HIP was not seen in all tumor types.

Xenotransplantation of PC3 or A549 cells into immunocompromised mice is a commonly used animal model for prostate or lung cancer, respectively. Extensive neovascularization occurs in the resulting tumors, and it has been demonstrated that angiogenesis inhibitors slow their growth [30,31]. The discovery that vWF-normalized mouse HIP mRNA levels were markedly decreased in A549 tumors and undetectable in all of the PC3 tumors we examined gives further weight to the hypothesis that decreased HIP expression is associated with tumor angiogenesis.

Angiogenesis has been well-characterized as a prerequisite to continued tumor growth and metastasis [16,17,32], and many approaches have been developed to attempt to control the progression of cancer by inhibiting angiogenesis [32,33]. A more complete analysis of the gene expression changes in endothelial cells during developmental and pathological processes should help to identify additional signaling pathways that are altered and that could be manipulated to control angiogenesis and disease progression. The identification of Shh as an angiogenic signaling molecule [7], along with the characterization of HIP as an inhibitor of Shh that is downregulated in certain tumors, strongly suggests that inhibition of the Shh pathway may be of therapeutic use in various cancers that rely on angiogenesis for their continued growth.

## Conclusions

We have shown that HIP is expressed predominantly in EC but at low or undetectable levels in other cell types, and that the high expression of HIP in EC may be responsible for their inability to respond to Shh. Expression of HIP in TM3 cells attenuated their response to Shh, demonstrating that HIP antagonizes Hh signaling when expressed in the responding cell. The reduction of HIP in a variety of human tumor samples as well as PC3 and A549 xenograft tumors, and in EC undergoing tube formation on Matrigel, suggests that modulation of HIP may play a role in tumor angiogenesis.

## Competing interests

None declared.

## Authors' contributions

CLO participated in experimental design and carried out CM production and assays, tissue and RNA processing, Q-RT-PCR, Western blot, and cancer array hybridization. PPH participated in the design of the study and performed tube formation assays, tissue and RNA processing, Q-RT-PCR, and Western blot. JG participated in initial characterization of HIP and provided supporting data. GMR was involved in the planning and organization of the study and reviewed the manuscript. ARB initiated, planned, and supervised the entire study.

## Pre-publication history

The pre-publication history for this paper can be accessed here:



## Figures and Tables

**Table 1:** HIP mRNA expression in human normal vs. tumor tissues.

	HIP/vWF*		
		
**Tissue**	**Normal**	**Tumor**	**Tumor/normal**
Liver 1	0.107	0	0
Liver 2	2.357	0	0
Kidney 1	0.306	0.051	0.17
Kidney 2	0.272	0.299	1.10
Breast	0.051	0.014	0.27
Lung 1	0.018	0.002	0.11
Lung 2	0.026	0.779	29.97

*Values are HIP mRNA expression normalized to that of vWF.

**Table 2:** Fold change in HIP for human tumor vs. normal tissues.

**Tissue**	**Sex**	**Race**	**Age**	**Tumor additional information**	HIP tumor/normal*
Liver	M	C	59	Primary cancer of the liver	0.41
	M	C	36	Primary cancer of the liver	0.06
	M	C	58	Primary cancer of the liver	0.03
					**HIP decreases in 3/3**
Stomach	M	C	63	Adenocarcinoma	0.16
	M	C	57	Gastric cancer	1.61
	M	C	47	Gastric cancer	0.24
	M	C	65	Adenocarcinoma	0.21
	M	C	61	Adenocarcinoma	0.24
	M	C	50	Mucinous adenocarcinoma	0.53
	F	C	72	Adenocarcinoma	0.68
	F	C	67	Adenocarcinoma	0.37
	M	C	75	Adenocarcinoma	0.03
	F	C	68	Adenocarcinoma	0.21
					**HIP decreases in 9/10**
Colon	F	C	67	Adenocarcinoma	0.25
	F	C	58	Tubulovillous adenoma	0.23
	F	C	43	Adenocarcinoma	0.85
	F	C	69	Adenocarcinoma	1.34
	F	C	35	Adenocarcinoma	0.41
	M	C	58	Adenocarcinoma	0.4
	M	C	63	Adenocarcinoma	0.24
	F	C	73	Adenocarcinoma	0.16
	F	C	65	Adenocarcinoma	1.68
	F	C	65	Adenocarcinoma	0.14
					**HIP decreases in 8/10**
Rectum	F	A	44	Mucinous adenocarcinoma	0.25
	M	C	68	Adenocarcinoma	1.1
	F	C	49	Adenocarcinoma	0.18
	F	C	59	Adenocarcinoma	0.22
	F	C	53	Mucinous adenocarcinoma	0.08
	F	C	70	Adenocarcinoma	1.05
	M	C	42	Adenocarcinoma	0.79
	F	C	71	Adenocarcinoma	0.59
	F	C	59	Adenocarcinoma	0.15
	M	C	43	Adenocarcinoma	0.56
					**HIP decreases in 8/10**
Lung	F	C	69	Bronchiolo-alveolar adenocarcinoma	6.45
	M	C	72	Central cancer of the right lung	0.36
	M	C	66	Central cancer of the middle lobe of rt lung	0.23
	M	C	51	Peripheral cancer of the upper lobe of rt lung	0.45
	M	C	63	Squamous cell carcinoma, keratinizing type	5.49
	M	C	62	Squamous cell carcinoma, keratinizing type	0.19
	M	C	67	Squamous cell carcinoma	0.4
	M	C	71	Squamous cell carcinoma, keratinizing type	0.95
	M	C	65	Adenocarcinoma	4.9
	M	A	52	Squamous cell carcinoma	7.08
					**HIP decreases in 5/10**

*HIP tumor/normal value is the vWF-normalized quantity of HIP for the tumor, divided by the vWF-normalized quantity of HIP for the corresponding normal tissue.
